# Neural Pathways Linking Autonomous Exercise Motivation and Exercise-Induced Unhealthy Eating: A Resting-State fMRI Study

**DOI:** 10.3390/brainsci14030221

**Published:** 2024-02-27

**Authors:** Ying Ling, Jinfeng Han, Yicen Cui, Wei Li, Hong Chen

**Affiliations:** 1 Faculty of Psychology, Southwest University, Chongqing 400715, China; ly000127@email.swu.edu.cn (Y.L.); hanjinfengpsy@126.com (J.H.); yicenc97@163.com (Y.C.); liweiswu@163.com (W.L.); 2 Key Laboratory of Cognition & Personality, Ministry of Education, Southwest University, Chongqing 400715, China

**Keywords:** autonomous motivation, exercise, unhealthy foods licensing, resting-state fMRI

## Abstract

Background: Unhealthy food compensation following exercise contributes to the failure of exercise for weight loss. Autonomous exercise motivation is a protective factor against exercise-induced unhealthy foods licensing (EUFL). However, the neural mechanism of exercise-specific autonomous motivation and how these neural correlates link to EUFL remain uncertain. Methods: This study explored the resting-state brain activity (i.e., amplitude or fractional amplitude of low-frequency fluctuations (ALFF/fALFF) and regional homogeneity (ReHo)) and seed-based functional connectivity (rsFC) of autonomous exercise motivation among 223 (72.3% female) healthy young adults. Autonomous exercise motivation and EUFL were measured by self-report measurements. Results: Results across resting-state indices and rsFC analysis show that autonomous exercise motivation was robustly associated with activity and connectivity within the cerebellum posterior lobe (PCB), middle frontal gyrus (MFG), and middle occipital gyrus (MOG). Specifically, the PCB acted as a hub, connecting the frontal and occipital lobes. Moreover, higher autonomous exercise motivation indirectly predicts reduced EUFL through enhanced activity in the MFG and connectivity of PCB–MOG. Conclusions: Neural substrate for enhanced conflict awareness and motor control may explain the protective effect of autonomous exercise motivation on post-exercise unhealthy eating. Enhancement of these functions could help regulate post-exercise eating and improve the effectiveness of exercise for weight loss.

## 1. Introduction

Unhealthy food eating following exercise can easily overcompensate for exercise-induced acute energy deficits [1,2,3], which contributes to failure when relying solely on exercise for weight loss [2,4,5]. Additionally, an unhealthy dietary structure is a significant contributor to chronic diseases, including cardiovascular diseases and diabetes (World Health Organization, 2003). Post-exercise unhealthy food eating varies widely among individuals and cannot be solely attributed to physiological factors [4,5]. Psychological factors, notably autonomous exercise motivation, have been suggested to play a significant role in post-exercise eating regulation [6,7]. More than just eating regulation, autonomous exercise motivation is linked to multiple health behavior improvements [8]. Thus, the present study aims to explore the internal mechanism through which autonomous exercise motivation influences post-exercise unhealthy eating behaviors by providing neurological evidence. The findings of our study may hold practical implications for the development of exercise and multiple health behavior intervention programs.

Compensation of unhealthy food post exercise is more likely to be a deliberate eating behavior rather than an obligatory metabolic response [6,7]. For instance, research noted that post-exercise energy ‘compensators’ showed a stronger preference for high-fat sweet foods compared with ‘non-compensators’, correlating this preference with increased caloric intake [1]. Individuals tend to consume more energy, primarily driven by high-calorie foods, after being informed of higher energy expenditure during exercise, because they view exercise-induced energy expenditure as a greater “license” to indulge in unhealthy but palatable foods [9]. According to the compensatory beliefs (CBs) model, compensating unhealthy foods after exercise may be rooted in the belief that the negative consequences of one behavior (e.g., feeling stressful during exercise) can be compensated for by engaging in another pleasurable behavior (e.g., eating hedonic foods) [10]. In this context, exercise behavior induces a “license” for hedonic eating, particularly among individuals who view unhealthy foods as a form of reward.

Recent studies have drawn attention to the positive effect of autonomous exercise motivation on exercise-induced unhealthy foods licensing [6,11]. According to self-determination theory (SDT) [8,12], exercise motivation can be categorized into autonomous motivation and controlled motivation. Autonomous exercise motivation is driven by the inherent interest, choice, or enjoyment of exercise behavior (called intrinsic motivation); or the full internalization of extrinsic motivation (called integration regulation) [12]. Extrinsic motivation refers to actions motivated by external outcomes, such as body shaping [12]. Meanwhile, insufficient internalization of extrinsic motivation (i.e., external regulation or introjected regulation) is identified as controlled motivation (relatively low autonomous motivation) [12]. The promotion of internalization requires external social support and nutrients [8,12].

Numerous studies have found that higher autonomous motivation for exercise is associated with more fruit and vegetable intake [13], more intuitive eating [14], fewer unhealthy eating behaviors/habits [15], and less unhealthy foods licensing after acute exercise [16,17,18,19]. The reviews suggest that individuals with autonomous motivation would activate fewer CBs post-exercise. First, people who exercise for autonomous motivation have already obtain positive instrumental and affective values in exercise experience and have no need to activate CBs [10]. Second, actions motivated by autonomous motivation hold a greater sense of self-control in goal-directed behaviors, including resisting the hedonic food temptation that might hinder their goals [6,10]. Behavioral studies have proven that affective experiences during exercise [20], self-control resources [21,22,23], and attitude toward foods [1,3] are influential factors in the relationship between autonomous exercise motivation and post-exercise unhealthy food eating, but the neural mechanism is poorly understood.

Though the neural basis of autonomous motivation has recently received attention [24,25,26], the neural exploration of exercise-specific autonomous motivation is significantly lacking. Reviews have outlined the way in which the neural basis of autonomous motivation mainly involves the striatum, orbitofrontal cortex (OFC), insula, and anterior cingulate cortex (ACC), contributing to the regulation of reward, emotion, and cognition processing [24,25,26]. These neural functions align precisely with the characteristics of autonomously motivated actions, being curiosity-driven, exploratory, self-determined, and goal-oriented [12]. Additionally, previous research has indicated a clear genetic basis for intrinsically motivated exercise [27], suggesting a potential neural mechanism for exercise-specific autonomous motivation. A recent review has suggested that regions associated with reward, such as the insula, are activated when exposed to exercise-related stimuli or within physically active groups [28]. Additionally, a functional near-infrared spectroscopy (fNIRS) study found that increased activation of the superior frontal gyrus (SFG), responsible for executive control, during walking was linked to a higher level of self-determined motivation for exercise [29]. Moreover, a neurobiological study has indicated that autonomous exercise motivation is positively associated with the availability of dopamine D2/3-receptor in the SFG and middle frontal gyrus [30]. Taken together, brain regions responsible for reward and cognition, such as the insula and the frontal lobes, may underlie the neural mechanism of autonomous exercise motivation.

The current research has predominantly concentrated on activating autonomous exercise motivation for specific tasks, such as imaging brisk walking or viewing exercise-related stimuli [28,29]. However, this approach may fail to capture the comprehensive neural mechanism underlying autonomous exercise motivation beyond these specific task behaviors. Resting-state functional magnetic resonance imaging (rs-fMRI) technology explores human behavior-related neural activity by detecting the spontaneous blood oxygen level-dependent (BOLD) signals in the brain [31,32,33]. Rs-fMRI technology not only offers advantages in data collection, particularly for specific populations (e.g., individuals with low intelligence), but also enables the investigation of whole-brain, multifunctional interactions that extend beyond the scope of task-based fMRI [33]. The amplitude of low-frequency fluctuations (ALFF), fractional amplitude of low-frequency fluctuations (fALFF), and regional homogeneity (ReHo) are effective and widely applied methods for evaluating resting-state regional brain activities [31,33]. Furthermore, functional connectivity (rsFC) analysis is utilized as a technique to explore collaborative relationships between two functional brain regions with the assistance of linear temporal correlation [33]. Seed-based rsFC analysis stands out as the earliest and most mature method for investigating functional connectivity in the brain [33]. Therefore, we employ three resting-state brain indices (ALFF/fALFF/ReHo) and seed-to-voxel rsFC analysis to address the following two research areas: (1) the potential neural correlates of autonomous exercise motivation, and (2) how these neural correlations are linked to exercise-induced unhealthy foods licensing.

## 2. Methods

### 2.1. Participants and Procedures

Ethics clearance was obtained from the university research ethics board prior to all data collection. The study was conducted in accordance with the Declaration of Helsinki (WMA 2000; Bošnjak 2001; Tyebkhan 2003). Participants were recruited from various online communities of a university in Chongqing, China. The prior sample size was calculated using G*power 3.1 calculator. A minimum sample size of 89 was required with a power of 0.95, *p*-level of 0.05, and a medium effect size of 0.15. For the initial screening (*n* = 226), participants were asked to truthfully fulfill the following inclusion criteria: no limitation to physical activity, absence of a current or history of neurological or psychiatric disorders, generally healthy (no use of psychoactive medications and no other chronic diseases), high school degree or higher education, and Chinese as the primary language. One participant was excluded for having either a current eating disorder or a history of one.

Upon arriving for the experimental session, each participant read and signed the informed consent form. They were then assigned to complete the self-report measures, and their height and weight were also measured. After completing the self-report session, participants were required to engage in a resting state fMRI scan. Before the scanning session, they had been told to avoid intense exercise for 24 h. After the fMRI scan, participants received monetary compensation for their participation. One participant was involved in exercise in the 24 h prior to the scan, and one participant’s fMRI scan data were not qualified for further analysis. Finally, a sample of *n* = 223 with 72.3% female, age range 17~26 years old (mean ± SD = 18.93 ± 1.02), and BMI from 16.22 to 31.31 kg/m^2^ (mean ± SD = 18.85 ± 0.93 kg/m^2^) was included in formal analysis.

### 2.2. Measures

#### 2.2.1. Body Mass Index (BMI)

A stadiometer and a balance beam scale (Detecto 339, Web City, MO, USA) was used to measure participants’ height and weight while dressed in light street clothing and without shoes. Then, BMI was calculated with the following formula: BMI = weight (kg)/height (cm)^2^.

#### 2.2.2. Autonomous Exercise Motivation

The Behavioral Regulation in Exercise Questionnaire-2 (BREQ-2) assesses an individual’s reasons for engaging in exercise [34]. It consists of five subscales, namely amotivation, external regulation, introjected regulation, identified regulation, and internal regulation. The Chinese-translated version of the BREQ-2 uses a 5-point scale from 0 (not at all true for me) to 4 (very true for me) and has been proven to have good psychometric properties [35,36]. McDonald’s omega index was employed as a reliability measure, as suggested by Goodboy and Martin [37], yielding the following internal consistency for each subscale: amotivation (ω = 0.79), external regulation (ω = 0.72), introjected regulation (ω = 0.80), identified regulation (ω = 0.73), and intrinsic motivation (ω = 0.92). The structural validity and reliability of measures were re-examined by confirmatory factor analysis (CFA) process as per previous studies [38]. The CFA results show that the structural validity and reliability of BREQ-2 was acceptable, as follows: χ^2^/df = 1.65, CFI = 0.95, TLI = 0.94, RMSEA = 0.05, SRMR = 0.06. Consistent with previous research [16,20], a relative autonomy index (RAI) was calculated to reflect the level of motivational autonomy, using the following formula: RAI = amotivation × (−3) + external × (−2) + introjected × (−1) + identified × (2) + intrinsic × (3). Higher RAI scores indicate greater autonomous motivation relative to controlled motivation.

#### 2.2.3. Exercise-Induced Unhealthy Foods Licensing (EUFL)

Exercise-induced unhealthy foods licensing was measured by the exercise-snacking licensing scale (ESLS) [39]. The six-item ESLS was developed in order to measure exercise-induced reward licensing of unhealthy snacks/drinks [39]. In order to investigate exercise-induced unhealthy foods licensing, the word “snacks” in relative items was replaced by “foods” in the present study. An example of an item is as follows: “After engaging in exercise, I feel that I can reward myself with unhealthy foods.” Participants responded on a 7-point scale ranging from 1 (strongly disagree) to 7 (strongly agree). A higher score reflects greater licensing of unhealthy foods/drinks following exercise. In the study, the internal consistency of these two subscales were both ω = 0.88. The CFA results show that the structural validity and reliability of ESLS was acceptable, as follows: χ^2^/df = 1.85, CFI = 0.99, TLI = 0.99, RMSEA = 0.06, SRMR = 0.02.

#### 2.2.4. Image Acquisition

A total of eight minutes of rs-fMRI scanning for each participant was performed using a 3-T Trio scanner (Siemens Medical, Erlangen, Germany). A gradient echo planar imaging sequence was used to obtain the functional image of the resting state. The scanning parameters were as follows: repetition time (TR) = 2000 ms; echo time (TE) = 30 ms; slices = 62; slice thickness = 2 mm; field of view (FOV) = 224 × 224 mm^2^; flip angle = 90°; resolution matrix = 112 × 112; voxel size = 2 × 2 × 2 mm^3^; and phase encoding direction was PC >> AC. Each section contained 240 volumes. The high-resolution T1-weighted structural images were also acquired as anatomical references for the functional scans (parameters: TR = 2530 ms; TE = 2.98 ms; FOV = 256 × 256 mm^2^; flip angle = 7°; base resolution = 256 × 256; slices = 192; voxel size= 1.5 × 1.5 × 1.5 mm^3^; and phase encoding direction = AC >> PC). During the scanning process, each participant was asked to remain still and relaxed, not to open his/her eyes, and to not think of anything deliberately. Foam pads and earplugs were used to reduce head motion and scanning noise.

#### 2.2.5. Image Data Preprocessing

The publicly available CONN functional connectivity toolbox (version 20.b; https://www.nitrc.org/projects/conn (accessed on 1 December 2020)) in conjunction with Statistical Parametric Mapping (SPM12) software (http://www.fil.ion.ucl.ac.uk/spm (accessed on 1 October 2014)), was used to perform all preprocessing steps on all collected neuroimaging data. To ensure a stable longitudinal magnetization state, initial images from the first 10 volumes during the resting-state scan were excluded. The subsequent preprocessing steps were as follows: (a) the images of each subject were first corrected for the slice timing to reduce differences in acquisition time between the slices within the scan, and then realigned to eliminate the influence of head motion; (b) the realigned images were spatially normalized to the Montreal Neurological Institute (MNI) space with a resolution voxel size of 2 × 2 × 2 mm^3^; (c) spatial smoothing was applied with a 6 mm full width at a half-maximum Gaussian kernel; (d) using the anatomical component-based correction method (aCompCor) [40,41], noise signals, such as signals from the cerebrospinal fluid and white matter (WM), and movement parameters were removed from the images as confounding variables [42,43]; (e) bad time points were considered as regressors and were defined by volumes with the mean framewise displacement (FD) power > 0.5 mm, as well as the two succeeding volumes and one preceding volume to reduce the spillover effect of head motion [44]; finally, (f) a bandpass temporal filtering (0.008–0.09 Hz) was used to remove the effects of very low-frequency drifts and high-frequency noises. For all participants, no head motion in any direction was >3 mm or rotation in any axis >3° during scanning, FD values did not exceed 0.50.

### 2.3. Data Analysis

#### 2.3.1. Neural–Behavior Correlation Analyses

We explored the resting-state brain activity related to autonomous exercise motivation using the ALFF, fALFF, and ReHo indices. All indices were calculated applying the DPARSF toolbox [45]. Amplitude of low-frequency fluctuations (ALFF) is an effective method for assessing voxel-wise BOLD signal dynamic characteristics in the low-frequency range (0.01–0.08 Hz) and has been shown to have high test-retest reliability [46]. The fractional amplitude of low-frequency fluctuations (fALFF) value is the ratio of low-frequency amplitude to the total amplitude [46]. To calculate fALFF, ALFF values are computed first. The specific steps involve transforming the time courses in each voxel that was transferred to the frequency domain. After calculating the square root of each frequency in the power spectrum, the mean square root was calculated in a low-frequency range (0.008–0.09 Hz); the result of this calculation becomes the value of ALFF. The fALFF is then computed as a fraction, with amplitudes in a low-frequency range (0.01–0.08 Hz) divided by the amplitudes throughout the frequency range (0.01–0.25 Hz).

Regional homogeneity (ReHo) measures the consistency of the time series between each voxel and its neighboring voxels, indicating the local consistency property of that voxel [47]. In contrast with ALFF/fALFF, which focuses on the intensity of resting-state activity, ReHo is more dedicated to the coherence and centrality of regional activity [47]. ReHo employs Kendall’s concordance coefficient (KCC), ranging from 0 to 1, to assess the local consistency of each voxel with its adjacent 26 voxels in the same time series. The ReHo value for each voxel is then normalized by dividing it by the average ReHo across the entire brain.

A whole-brain correlation analysis was conducted to identify the relationships between regional spontaneous brain activity (using ALFF/fALFF/ReHo) and autonomous exercise motivation (using RAI) applying DPABI software (version 6.0; http://rfmri.org/dpabi (accessed on 1 May 2021)) [48]. Sex, age, and FD are covariates for their potential associations with brain activity. To infer the regions of significance, we used the Gaussian random field approach (GRF) [49] with the following settings: *p* < 0.05 at the cluster level, *p* < 0.005 at the voxel level, and dimensions of 61 × 73 × 61.

#### 2.3.2. Seed-to-Voxel rsFC Analysis

Applying the DPARSF toolbox, we conducted a seed-to-voxel rsFC analysis. First, we used brain regions that were significantly correlated with RAI as regions of interest (ROIs) and averaged the time series of all voxels for each ROI and other voxels in the whole brain. We then conducted a correlation analysis between ROIs and other voxels and gained their correlations maps at the participant level. Finally, we performed a correlation analysis using DPABI software to examine whether any specific correlations in maps were associated with RAI. Additionally, sex, age, and FD were controlled as covariates. The GRF approach was used for multiple comparisons correction (*p* < 0.05 at the cluster level and *p* < 0.005 at the voxel level).

#### 2.3.3. Mediation Model Analysis

To verify if the regional resting-state brain activity and/or connectivity mediate the association between autonomous exercise motivation and exercise-induced unhealthy foods licensing, we carried out a mediation analysis using the PROCESS macro in SPSS 22.0 (Model 4) [50]. In the mediation model, we took the autonomous exercise motivation (RAI as predictor) as independent variable, potential regional brain activity or connectivity as mediator variable, and exercise-induced unhealthy foods licensing as dependent variable in the model. The significance of the mediating effect was assessed using a bootstrapping method with 1000 iterations. If a 95% confidence interval (CI) does not contain zero, then the mediating effect is significant.

## 3. Results

### 3.1. The Correlations between Behavioral Variables

The descriptions and correlation results of behavioral variables are presented in Table 1. Sex (men = 0, women = 1) was negatively related to BMI (*r* = −0.16, *p* < 0.05) and RAI (*r* = −0.23, *p* < 0.01), and it was positively related to exercise-induced unhealthy foods licensing (*r* = 0.19, *p* < 0.01). Age was negatively related to exercise-induced unhealthy drink licensing (*r* = −0.14, *p* < 0.05). Autonomous exercise motivation (RAI) was negatively associated with exercise-induced unhealthy foods licensing (*r* = −0.15, *p* < 0.05). Exercise-induced unhealthy foods licensing was highly correlated with drink licensing (*r* = 0.88, *p* < 0.001). No other significant correlation was found among these variables.

### 3.2. Autonomous Exercise Motivation-Related Resting-State Brain Activity

**Figure 1:**
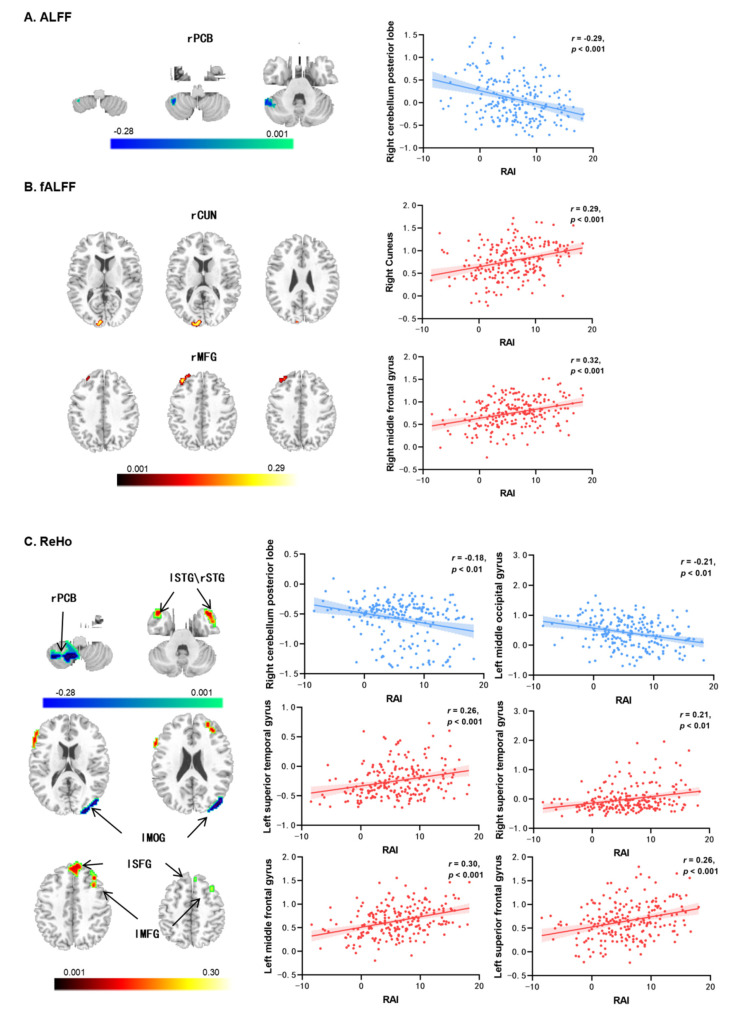
Resting-state brain activities associated with autonomous exercise motivation (regarding RAI as the predictor) controlled sex, age, and FD. (**A**) The correlations with ALFF; (**B**) the correlations with fALFF; (**C**) the correlations with ReHo. Notes: ALFF = amplitude of low-frequency fluctuations, fALFF = fractional amplitude of low-frequency fluctuations, ReHo = regional homogeneity, rPCB = right cerebellum posterior lobe, rCUN = right cuneus, lMFG/rMFG = left/right middle frontal gyrus, lSTG/rSTG = left/right superior temporal gyrus, lMOG = left middle occipital gyrus, lSFG = left superior frontal gyrus, RAI = relative autonomy index.

The results of the ALFF and fALFF analyses are presented in Table 2. There was a significant negative correlation between RAI and the ALFF in the right cerebellum posterior lobe (rPCB) (*r* = −0.29, *p* < 0.001; see Figure 1A). Additionally, RAI was positively correlated with the fALFF of the right cuneus (rCUN: *r* = 0.29, *p* < 0.001; see Figure 1B) and the right middle frontal gyrus (rMFG: *r* = 0.32, *p* < 0.001; see Figure 1B).

The results of the ReHo analysis are shown in Table 2 and Figure 1C. The autonomous exercise motivation (RAI) was significantly correlated with the ReHo values in six brain regions. Specifically, RAI exhibited a negative correlation with the right cerebellum posterior lobe (rPCB; *r* = −0.18, *p* < 0.01) and the left middle occipital gyrus (lMOG; *r* = −0.21, *p* < 0.01). On the other hand, RAI showed positive correlations with the left superior temporal gyrus (lSTG; *r* = 0.26, *p* < 0.001), right superior temporal gyrus (rSTG; *r* = 0.21, *p* < 0.01), left middle frontal gyrus (lMFG; *r* = 0.30, *p* < 0.001), and left superior frontal gyrus (lSFG; *r* = 0.26, *p* < 0.001).

### 3.3. Autonomous Exercise Motivation-Related Functional Connectivity

**Figure 2:**
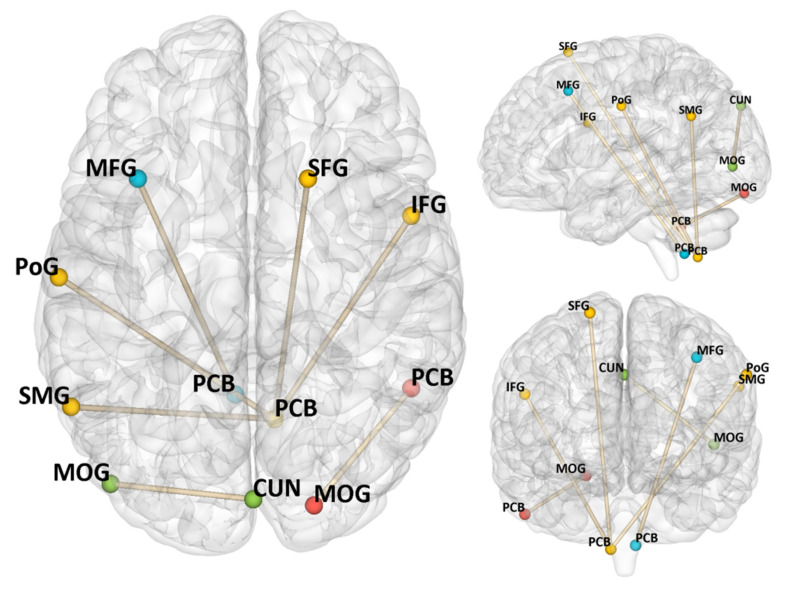
Resting-state functional connectivity associated with autonomous exercise motivation (regrading RAI as the predictor) controlled sex, age, and FD. Notes: PCB = cerebellum posterior lobe, CUN = cuneus, MFG = middle frontal gyrus, MOG = middle occipital gyrus, SMG = supramarginal gyrus, PoG = postcentral gyrus, IFG = inferior frontal gyrus, SFG = superior frontal gyrus, RAI = relative autonomy index.

The results of rsFC analysis are presented in Table 2 and Figure 2. The connection between the right PCB (found in ALFF analyses) and the right MOG was positively correlated with RAI (rPCB–rMOG: *r* = 0.27, *p* < 0.001).

With the right PCB (found in ReHo analyses) as a seed, its connections to the right inferior frontal gyrus (rIFG), the left supramarginal gyrus (lSMG), the left postcentral gyrus (lPoG), and the right SFG were significantly correlated with RAI (rPCB–rIFG: *r* = −0.26, *p* < 0.001; rPCB–lSMG: *r* = 0.27, *p* < 0.001; rPCB–rPoG: *r* = −0.26, *p* < 0.001; rPCB–rSFG: *r* = 0.25, *p* < 0.001). Additionally, the connection between the left MOG and the right cuneus (rCUN) was positively correlated with RAI (rMOG–rCUN: *r* = 0.31, *p* < 0.001), and the connection between left MFG and the left PCB was negatively correlated with RAI (lMFG–lPCB: *r* = −0.34, *p* < 0.001).

### 3.4. Mediation Model Results

**Figure 3:**
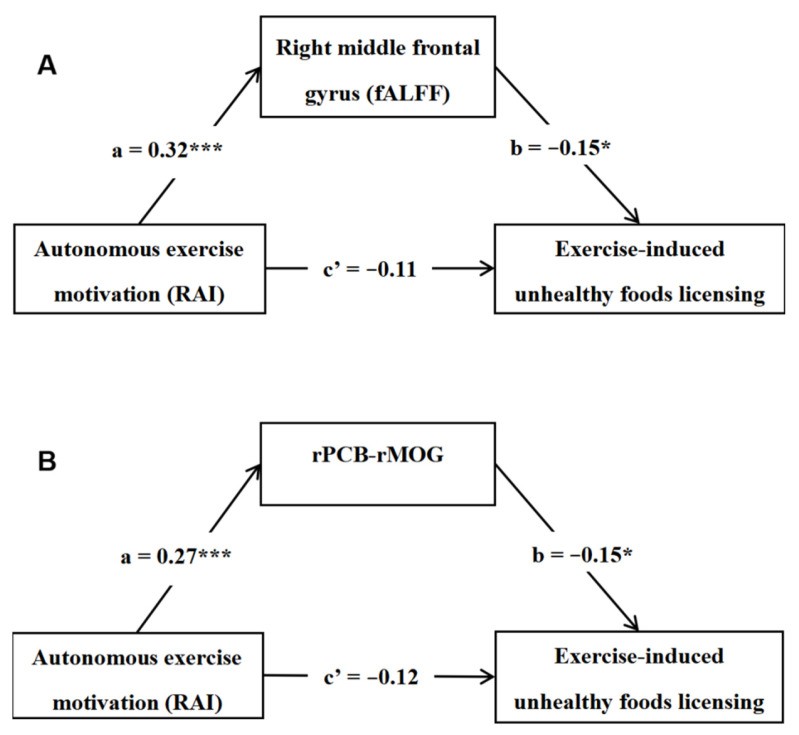
The fALFF in the right MFG (**A**) and the functional connectivity between the right PCB and the right MOG, (**B**) both totally mediated the relationship between autonomous exercise motivation and exercise-induced unhealthy foods licensing. Notes: RAI = relative autonomy index, fALFF = fractional amplitude of low-frequency fluctuations, r = right, PCB = cerebellum posterior lobe, MOG = middle occipital gyrus, * *p* < 0.05, *** *p* < 0.001.

To examine whether the resting-state brain activity/connectivity could mediate the relationship between autonomous exercise motivation (RAI) and exercise-induced unhealthy foods licensing (EUFL), we conducted a mediation analysis. Sex, age, and FD were also controlled as covariates. As depicted in Figure 3A, the fALFF in the right MFG totally mediated the relationship between RAI and EUFL (indirect effect = −0.05, 95%CI [−0.10, −0.01]). The mediating effect accounted for 29.76% variation of the total effect (c = −0.15, *p* < 0.05). In addition, as presented in Figure 3B, the rsFC between the right PCB and the right MOG also totally mediated this relationship (RAI → rPCB–rMOG → EUFL; indirect effect = −0.04, 95%CI [−0.09, −0.01]). And this mediating effect explained 25.08% variation of the total effect (c = −0.15, *p* < 0.05).

## 4. Discussion

This study primarily explores the resting-state brain activity and functional connectivity associated with autonomous exercise motivation and validated the associations of these neural correlations with post-exercise unhealthy foods licensing. The ALFF/fALFF/ReHo analyses consistently found that the neural correlates of exercise-specific autonomous motivation were robustly located in the cerebellum posterior lobe (PCB), middle frontal gyrus (MFG), and middle occipital gyrus (MOG, containing the cuneus). Further rsFC analyses revealed that the PCB acted as a crucial neural hub that linked the cerebellum to the frontal, occipital, and parietal lobes. Moreover, the resting-state activity in the right MFG and connectivity of PCB–MOG were determined to be the potential neural pathways linking autonomous exercise motivation and exercise-induced unhealthy foods licensing. These findings imply that the neural profiles of exercise-specific autonomous motivation may involve processes of salience detection, conflict monitoring, motor control, and emotion regulation, which explains, from a neural perspective, the protective effect of autonomous exercise motivation on post-exercise impulsive eating behaviors.

Employing ALFF and ReHo analyses, the present fMRI study found that autonomous exercise motivation (with RAI as the predictor) was negatively associated with the resting-state activity in the right PCB. The PCB is traditionally found to be involved in motor control, but accumulating evidence indicates that it may specifically engage in motivational, emotional, and cognitive aspects of motor function [51,52]. For instance, the PCB was extra activated during the motor task with verbal encouragement compared with the motor task without verbal encouragement [53]. These functions fit well with the regulation of autonomous motivation, which require an acute perception of environmental antecedents or any motivational context that is supportive of autonomous motivation (e.g., encouragement) and an effective internalization to self-determine behaviors [8,12]. Autonomous exercise motivation is well-proven to be associated with exercise engagement and adherence, as well as with the enrichment of experiences across exercise settings [8]. Hence, the inverse association observed between autonomous exercise motivation and resting-state activity in the PCB might indicate an improved involvement of the PCB in motor tasks, facilitating the processing of autonomy-supportive motivational context for enhanced motor control.

In further rsFC analyses, we found that the PCB acted as a crucial node connecting the frontal lobes (i.e., MFG, SFG), parietal lobe (i.e., SMG), and occipital lobes (i.e., MOG, CUN). These observations are in line with existent views stating that the cerebellum has rich connections with other brain areas to support the management of various inputs from these origins and feedback to other relevant brain areas in order to behave in an adaptive way [51,52]. For instance, a prior study has observed that the cerebellum hemispheres, frontal lobes, and parietal lobes are simultaneously strongly activated when imaging faster brisk walking [54]. Additionally, autonomous exercise motivation exhibits a positive correlation with various adaptive indicators of wellbeing and enriched experiences within sport and exercise conditions [8]. Thus, the rsFC of the PCB with other brain regions indicates effective communication between the body and the external environment, contributing to adaptive and enhanced goal-directed performance, such as increased persistence in exercise. Notably, the PCB may be the unique neural marker of exercise-specific autonomous motivation, as it has so far not appeared in neural studies of general autonomous motivation [24,25,26].

Consistent with previous neuroimaging studies [24,25,26,30,55], autonomous exercise motivation has been found to be positively associated with the resting-state activity in the frontal lobe (i.e., MFG, SFG). The frontal lobes mediate cognitive control and motivation to shape human behavior [56]. Specifically, the MFG plays a role in suppressing motivational biases when they conflict with instrumental goals [57]; while the SFG plays a role in the context of reward and effort expenditure [56,58]. Previous studies have suggested that the significance of the frontal lobe is in its adaptation to failures within autonomous motivation conditions, resulting in improved performance [24,55]. Moreover, a prior study has found a concentration of dopamine receptors in the MFG and SFG [30]. Dopamine is the basic neural substrate of reward systems [30]. Thus, these findings suggest that autonomous exercise motivation may reflect a neural mechanism encompassing reward-governing, conflict monitoring and cognitive control.

The present study has found that brain activity in the occipital lobes, such as MOG and CUN, is significantly associated with autonomous exercise motivation. The MOG is involved in various functions, such as visual perception, body shape processing, and auditory organization [59,60]. Additionally, the CUN, a region adjacent to the MOG, plays an important role in the integration of visual information, working memory and cognitive processing [61]. Actions motivated autonomously are curiosity-driven exploratory activities, and require sufficient information seeking mechanisms [62]. Thus, the engagement of the occipital lobes may meet the information seeking and processing needs for exercising under autonomous motivation.

The second aim of this study was to find the neural pathways connecting autonomous exercise motivation and unhealthy eating after exercise. First, we found that the fALFF in the right MFG totally mediated this relation. As mentioned above, the MFG is involved in suppressing motivational biases when they conflict with goals [57]. Post-exercise unhealthy foods licensing is in conflict with goals under autonomous exercise motivation, such as exercise for health. Thus, enhanced function in the MFG may help individuals resist the temptation of hedonic foods following exercise. Previous studies have also suggested that the MFG is involved in eating regulation [63,64]. Second, the present study has also found the rsFC of PCB–MOG totally mediated this relationship. Reviews indicate that the role of PCB in eating is to respond to prediction errors arising across the motor, cognitive, and affective domains [65]. In collaboration with the visual searching function of the MOG, the PCB–MOG may reflect enhanced feed-forward predictions used to compare expected outcomes to sensory feedback. Food consumption is significantly influenced by expectation [65]. Therefore, sufficient outcome predictions and conflict control may help autonomously motivated individuals suppress hedonic expectation of unhealthy foods and adherence to goal-directed behaviors.

Some limitations should be noted when interpreting these results. First, the present study only used RAI as the predictor of autonomous exercise motivation. Multiple measurements of autonomous exercise motivation, such as self-report intrinsic motivation for exercise or behavioral autonomous exercise motivation, should be investigated in order to reveal a more comprehensive neural mechanism. Second, post-exercise unhealthy eating behavior was measured by a self-report method, which lacks ecological validity. Real eating behaviors following exercise can be studied in further neuroimaging studies. Third, due to the lack of available neurological evidence, this study adopted an exploratory cross-sectional study design, studies using longitudinal study designs, etc. should also be implemented in the future. Furthermore, multiple neural methods, such as a priori target ROI setting approach, task-based fMRI and voxel-based morphometry (VBM) analysis, are required for further research in order to increase the reproducibility of brain-wide association studies. Finally, the generalizability of our findings may be constrained as the participants were chosen from a small age range and highly homogeneous group of healthy Chinese university students. Further research should investigate groups among a wider range of ages and other social statuses. Additionally, it is necessary to focus on individuals with severe exercise-induced unhealthy compensatory eating behaviors.

## 5. Conclusions

As the first study to explore the neural mechanism of exercise-specific autonomous motivation, the present study neurologically validated the profiling of self-determination theory for autonomous exercise motivation. Specifically, the PCB may be a unique neural marker of autonomous motivation in the exercise domain. For practical implications, the neural findings provide new referential value for intervention programs. In addition to behavioral interventions (i.e., need-supportive intervention), neuromodulation strategies (e.g., transcranial magnetic stimulation (TMS), transcranial direct current stimulation (tDCS), and neurofeedback) may also be applicable to autonomous exercise motivation interventions. Furthermore, the neural pathways linking autonomous exercise motivation and exercise-induced eating behavior suggest that the synergistic effects of autonomous exercise motivation may promote multiple health behaviors. Future research should focus on the behavioral and cognitive consequences for these neural marker alterations to determine whether these neuroimaging findings could guide the development of targeted prevention and intervention aimed at enhancing autonomous exercise motivation or even multiple health behaviors.

## Figures and Tables

**Table 1:** The correlations between behavioral variables (*n* = 223).

Indices	Mean	SD	1	2	3	4	5	6
1. Sex	-	-	-					
2. Age	18.85	0.94	−0.01	-				
3. BMI	21.13	3.30	−0.16 *	0.07	-			
4. FD	0.09	0.04	−0.10	−0.04	0.10	-		
5. RAI	5.99	5.28	−0.23 **	0.02	−0.01	−0.01	-	
6. EUFL	11.16	4.05	0.19 **	−0.11	−0.05	−0.05	−0.15 *	-
7. EUDL	11.50	4.39	0.11	−0.14 *	−0.05	−0.01	−0.08	0.88 ***

Notes: * *p* < 0.05, ** *p* < 0.01, *** *p* < 0.001; BMI = body mass index, FD = framewise displacement, RAI = relative autonomy index, EUFL/EUDL = exercise-induced unhealthy foods/drink licensing.

**Table 2:** The resting-state brain activity related to autonomous exercise motivation (*n* = 223).

Brain Regions	H	Peak MNI Coordinates			Peak T	Voxel Size
		X	Y	Z		
**Correlations with ALFF**						
Cerebellum posterior lobe (PCB)	R	54	−50	−36	−4.27	268
**Correlation with fALFF**						
Cuneus (CUN)	R	10	−96	12	3.95	167
Middle frontal gyrus (MFG)	R	38	34	44	4.43	82
**Correlation with ReHo**						
Cerebellum posterior lobe (PCB)	R	9	−60	−54	−3.79	141
Superior temporal gyrus (STG)	L	−42	12	−24	4.43	185
Superior temporal gyrus (STG)	R	42	18	−36	3.95	320
Middle occipital gyrus (MOG)	L	−45	−81	0	−4.27	218
Middle frontal gyrus (MFG)	L	−36	18	45	4.76	114
Superior frontal gyrus (SFG)	L	−6	48	36	4.60	99
**Seed-based Functional Connectivity**						
**Seed: PCB_R (ALFF)**						
Middle occipital gyrus (MOG)	R	22	−88	−16	3.76	813
**Seed: PCB_R (ReHo)**						
Inferior frontal gyrus (IFG)	R	54	6	26	3.63	181
Supramarginal Gyrus (SMG)	L	−58	−56	30	−4.07	199
Postcentral gyrus (PoG)	R	−62	−14	36	3.85	195
Superior frontal gyrus (SFG)	R	20	18	68	−4.17	183
**Seed: MOG_L (ReHo)**						
Cuneus (CUN)	R	2	−86	36	5.36	3813
**Seed: MFG_L (ReHo)**						
Cerebellum posterior lobe (PCB)	L	−4	−52	−52	−4.85	282

Notes: The threshold for significant regions was set at *p* < 0.05 at the cluster level and *p* < 0.005 at the voxel level, two-tailed. H= hemisphere, ALFF = amplitude of low-frequency fluctuations, fALFF = fractional amplitude of low-frequency fluctuations, ReHo = regional homogeneity, R/L = right/left.

## Data Availability

Due to the sensitive nature of the questions asked in this study, survey respondents were assured raw data would remain confidential and would not be shared.
